# Prognostic Value of Traditional and Optimised Vital Sign Thresholds for Risk of Serious Adverse Events in Continuously Monitored Hospitalised Patients

**DOI:** 10.3390/s26175399

**Published:** 2026-08-26

**Authors:** Nikolaj Aagaard, Markus Harboe Olsen, Jesper Mølgaard, Katja Kjær Grønbæk, Mikkel Elvekjær, Eske K. Aasvang, Christian S. Meyhoff

**Affiliations:** 1 Department of Anaesthesia and Intensive Care, Copenhagen University Hospital—Bispebjerg and Frederiksberg, 2400 Copenhagen, Denmark; markus.harboe.olsen@regionh.dk (M.H.O.); katja.groenbaek@ward247.com (K.K.G.); mikkel.elvekjaer.02@regionh.dk (M.E.); christian.sylvest.meyhoff@regionh.dk (C.S.M.); 2 Department of Neuroanesthesiology, The Neuroscience Centre, Copenhagen University Hospital—Rigshospitalet, 2100 Copenhagen, Denmark; 3 Department of Anaesthesia, Centre for Cancer and Organ Diseases, Copenhagen University Hospital—Rigshospitalet, 2100 Copenhagen, Denmark; jesper.moelgaard@regionh.dk (J.M.); eske.kvanner.aasvang.01@regionh.dk (E.K.A.); 4 Department of Clinical Medicine, University of Copenhagen, 2200 Copenhagen, Denmark

**Keywords:** continuous vital sign monitoring, wireless biosensors, acute complications, serious adverse events, surgical patients, medical patients

## Abstract

Technological advances enable continuous vital sign monitoring with wireless biosensors in general wards. However, traditional thresholds used in routine monitoring do not effectively identify serious adverse events (SAEs). This study aimed to assess the prognostic ability of combinations of threshold severity and duration in continuous vital sign monitoring for SAEs in acute medical and surgical patients. Data on heart rate (HR), respiratory rate (RR), and peripheral oxygen saturation (SpO_2_) were collected from four prospective observational studies and two randomised controlled trials. The primary outcome was an SAE within 30 days. Traditional thresholds were based on the National Early Warning Score, and optimised thresholds were derived by identifying threshold values and durations with the highest area under the receiver operating characteristic curve (AUROC) for any SAE and specific SAE types. Of 1492 patients, 1077 were included in HR and RR analyses and 761 in SpO_2_ analyses; 30% experienced an SAE. For any SAE, traditional thresholds had AUROC values of 0.47–0.50 (95% CI 0.43–0.54). Optimised thresholds also showed limited ability (AUROC 0.51, 95% CI 0.44–0.56). In contrast, 18 thresholds for specific SAE types had AUROC > 0.70; the highest AUROC was observed for HR > 104 beats/min for ≥36 min for upcoming respiratory SAEs (AUROC 0.82, 95% CI 0.74–0.90). Optimised thresholds showed low prognostic ability for any SAE but higher AUROC than traditional thresholds. Several thresholds demonstrated moderate prognostic ability for specific SAE groups and warrant validation.

## 1. Introduction

Delayed recognition of patient deterioration in hospital wards increases in-hospital mortality and ICU admissions [1]. As a result, routine manual vital sign monitoring at fixed intervals, such as the National Early Warning Score (NEWS), has been implemented [2]. No documented effect of NEWS on morbidity or mortality has been observed [3], although rapid response systems have been associated with improved clinical outcomes, emphasising the importance of effective escalation and response [4]. This may be partly due to deterioration occurring between observations, where continuous vital sign monitoring (CVSM) with biosensors measuring heart rate (HR), respiratory rate (RR), and peripheral oxygen saturation (SpO_2_) enables early detection, potentially facilitating timely treatment and prevention of further clinical decline [5,6].

Implementation of CVSM may decrease the risk of serious adverse events (SAEs) [7], but NEWS-based vital sign thresholds evaluated in our previous studies did not consistently predict subsequent SAEs when applied to CVSM [8,9]. Physicians consider both the severity and duration of vital-sign abnormalities when assessing patients, yet this temporal dimension is not incorporated into conventional monitoring systems, limiting their utility in acute care settings. Optimising threshold values and the duration spent outside these thresholds may improve prediction of SAEs, both by distinguishing patients with and without SAEs and by identifying changes preceding SAEs in affected patients. This is important, as a CVSM alert system that generates too many false or irrelevant alarms can contribute to alarm fatigue and increase the risk of overtreatment or unnecessary diagnostic procedures [10].

This study aimed to explore optimised vital sign thresholds that incorporated duration and severity of the deviations for prognostic ability of subsequent SAEs, when measured continuously in patients hospitalised for acute medical disease or after major surgery. The prognostic ability was investigated by calculating the area under the curve based on 24 h periods of CVSM. Our research group has previously used similar methodology to analyse continuous measurements of heart rate variability, a more novel CVSM-derived parameter, finding prognostic ability for specific SAE subtypes, including cardiovascular and respiratory SAEs [11]. We applied this approach to continuous measurements of HR, RR and SpO_2_, which are widely used in clinical practice to identify deteriorating patients [2], to quantify the prognostic value and identify relevant thresholds for alarms in CVSM systems. We hypothesised that optimised thresholds would have better prognostic ability than thresholds used in NEWS, and secondly that optimised thresholds would have even better prognostic ability for specific subtypes of SAEs.

## 2. Materials and Methods

### 2.1. Included Studies

Patients were included from four prospective observational studies (NCT03660501, NCT03491137, NCT04473001, and NCT04628858) and two randomised controlled trials (NCT04661748 and NCT04640415). All studies were approved by the Danish Data Protection Agency (P-2019-690) and regional ethics committee (H-20033246, H-20034555, H-18026653, H-17033535, H-20002220, and H-19086583). The research was conducted in accordance with the principles embodied in the Declaration of Helsinki and in accordance with local statutory requirements. All studies included adult patients after either acute medical admission or major surgery that provided written informed consent before enrolment. A full list of inclusion and exclusion criteria is provided in Supplemental Table S1.

### 2.2. Data Collection

Data were collected using the clinically validated CE- and FDA-approved Isansys Lifetouch (Isansys Lifecare, Abingdon, Oxfordshire, United Kingdom) and Nonin WristOx 3150 (Nonin Medical inc., Plymouth, MN, USA) [12,13] (Supplemental Figure S1). Isansys Lifetouch is an electrocardiographic (ECG) patch attached to the patient’s chests with two electrodes placed 15 cm apart over the anterior left aspect of the patient’s thorax at an angle of 45° with its base in the fourth intercostal space approximately 2 cm lateral of the sternum. The device recorded a single-lead ECG with HR and RR calculated from 10 s segments with a 1 min sampling frequency. The 10 s segment was chosen to extend device battery lifetime instead of a full 60 s ECG per minute. The RR measurements were derived from automatic calculations of changes in the single-lead ECG (R-peaks) during the respiratory cycle due to changing impedance of the thoracic cavity. Nonin WristOx 3150 is a wearable fingertip pulse oximeter measuring SpO_2_ with a sampling frequency of one per second. SpO_2_ measurements were only included if at least 45 s of each minute were reliable.

All devices transferred data directly via Bluetooth to a bedside gateway (Isansys Lifecare, Abingdon, Oxfordshire, United Kingdom) and afterwards through a secure hospital Wi-Fi connection to a dedicated hospital server. Data on HR and RR were automatically stored on the device when a patient was out of Bluetooth range and transferred when the connection was re-established. CVSM was initiated after informed consent and continued for up to 96 h during hospitalisation. Data obtained from CVSM in the intervention groups were available to the staff in the two randomised controlled trials.

### 2.3. Thresholds

Thresholds represent specific values for each vital sign. Thresholds for RR and HR were systematically investigated using 1-integer increments of breaths per minute (brpm) and beats per minute (bpm), respectively. SpO_2_ thresholds were examined with 1-percentage-point increments. The thresholds investigated ranged from 20 to 150 bpm for HR (130 thresholds in total), 4 to 40 brpm for RR (36 thresholds in total), and 30% to 100% (70 thresholds in total) for SpO_2_.

### 2.4. Exposures

**Figure 1:**
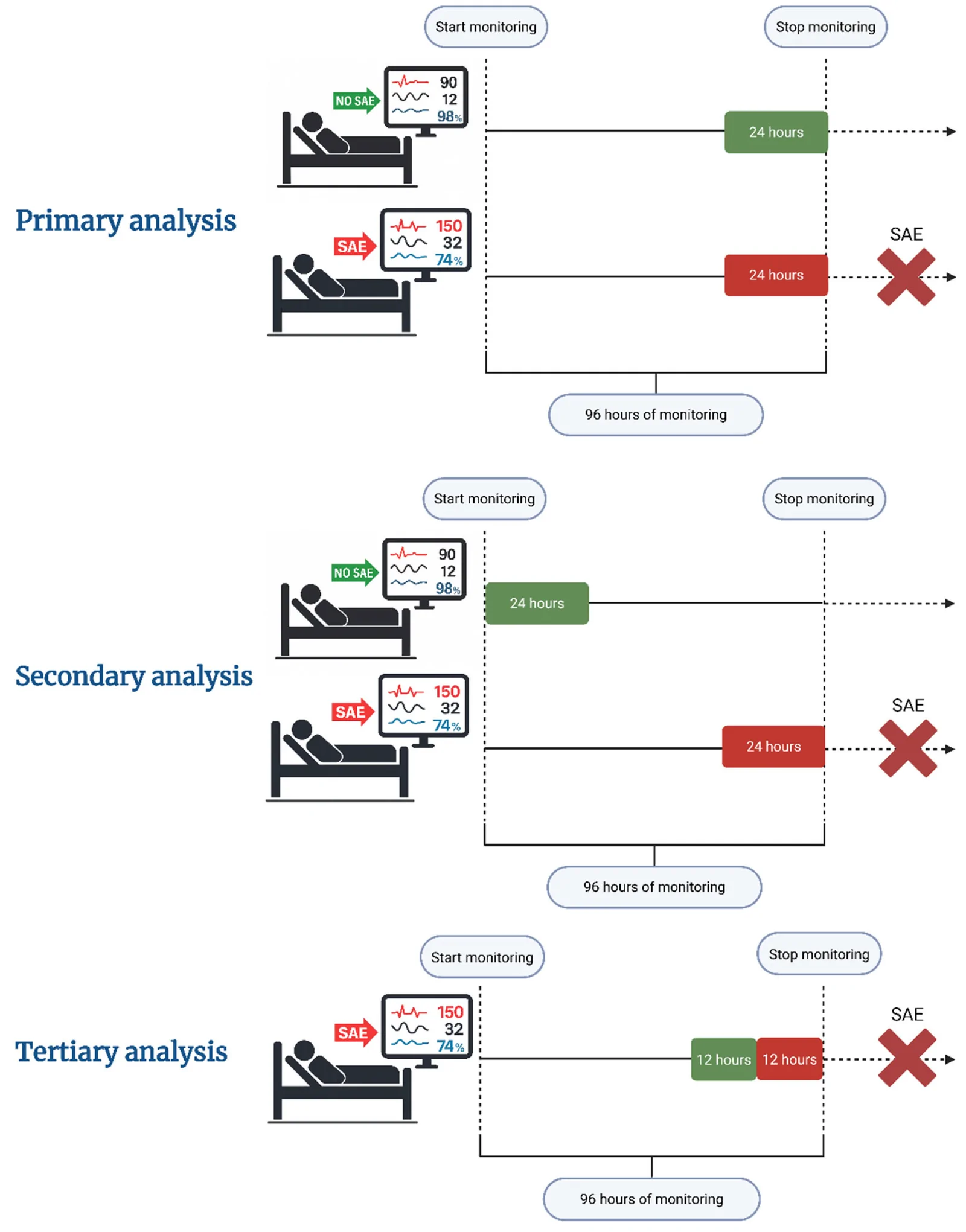
Overview of exposure periods and corresponding analyses. Legend: Created in BioRender. BioRender.com. SAE: serious adverse event. The primary analysis compared the final 24 h before an SAE with the final 24 h of monitoring in patients without an SAE. The secondary analysis compared the final 24 h before an SAE with the first 24 h of monitoring in patients without an SAE. Only patients with at least 12 h of monitoring for each respective vital sign within the 24 h periods of interest were included. The tertiary analysis included only patients with an SAE and compared the last 12 h before an SAE with the preceding 12–24 h. Only patients with at least 6 h of monitoring in both periods were included. Patients experiencing an SAE within the first 24 h of monitoring were excluded. If the SAE occurred during monitoring, the final 24 h preceding the event were used for analysis.

Exposure variables were accumulated duration in minutes outside specific thresholds for each vital sign (HR, RR, SpO_2_) during the last 24 h of measurement before the first SAE regardless of whether the complication occurred during monitoring, or up to 30 days after monitoring ended. If no SAE occurred, the last 24 h of vital sign measurements in each patient were used for the primary analysis, and the first 24 h of vital sign measurements were used in a secondary analysis. Only patients with at least 12 h of monitoring for each respective vital sign in the 24 h of interest were included in the analysis. We conducted these analyses to determine if changes in vital signs during monitoring had prognostic ability for SAE development when compared to a control group without SAE development. We evaluated different exposure periods in the control group to assess the impact on prognostic ability, an important analysis considering all participants were admitted for major surgery or acute medical conditions and a degree of vital sign deviation was expected. The tertiary analysis only included patients with an SAE. We compared the last 12 h of measurements before an SAE with the period measured 12–24 h prior to the SAE. Only patients with at least 6 h of monitoring time within both periods were included. This analysis aimed to investigate if vital sign deviations in the period leading up to an SAE compared to earlier monitoring had prognostic ability. Patients with SAEs within the first 24 h were excluded to ensure sufficient monitoring time for prognostic evaluation of SAEs and to avoid inclusion of pre-existing SAE or SAE related to initial treatment [14]. Only vital sign measurements preceding the first SAE were included to avoid bias of vital sign deviation due to the first SAE and the medical interventions to correct it. An overview of exposure periods and corresponding analyses is illustrated in Figure 1.

### 2.5. Outcomes

The primary outcome was the first occurring SAE of any type within 30 days. SAEs were defined as any medical life-threatening complications, resulting in death, hospitalisation, prolongation of existing hospitalisation, or significant disability according to the International Conference on Harmonisation-Good Clinical Practice guideline [15]. Secondary outcomes were all-cause mortality and non-fatal SAEs within the following classifications: cardiovascular, respiratory, infectious, neurologic, and other SAEs within 30 days. All outcomes were extracted from the electronic health records by physicians, based upon a standardised outcome manual including international defined criteria, such as acute renal failure, myocardial infarction, and sepsis [16,17,18].

### 2.6. Statistical Analysis

Descriptive statistics were used to analyse baseline characteristics and frequency of patients with and without SAEs. Categorical variables are presented as numbers with percentages and continuous variables as medians with interquartile ranges.

We calculated the area under a receiver operating characteristics curve (AUROC) and the corresponding 95% confidence interval (CI), using stratified bootstrapping, with the pROC-package [19]. The binary response variable was the occurrence of an SAE, and the continuous predictor variable was the accumulated duration outside each specific threshold. AUROC quantified the prognostic ability and was interpreted as representing ‘no better than chance’ (~0.50), low prognostic ability (0.50–0.70), moderate prognostic ability (0.70–0.90), and high prognostic ability (>0.90) [20].

Additional calculations of the optimal cut-off and corresponding sensitivity and specificity were performed for all thresholds. The optimal cut-offs were defined as the values of the predictor variable, specifically the duration in minutes outside the threshold, that maximised Youden’s Index, i.e., the highest combined sum of specificity and sensitivity [21]. This approach allowed the determination of a two-dimensional alarm structure that tested the optimal combination of threshold severity and duration outside the threshold. Optimised thresholds with durations >180 min were excluded to emphasise thresholds with potential clinical applicability as alarm thresholds. For traditional NEWS-based thresholds, an a priori cut-off of 60 min was used for HR > 130 bpm, RR > 24 brpm, RR < 9 brpm, SpO_2_ < 92%, and 5 min for HR < 41 bpm. An optimised approach for determining the cut-off was not applied, as these thresholds are predefined in clinical practice and currently not subject to data-driven adjustments. Unlike NEWS, the optimised thresholds evaluated individual vital signs rather than a composite score. Accordingly, we compared them with the corresponding individual NEWS-based thresholds, which are commonly used for alarm generation in CVSM systems [22]. As this was an exploratory analysis including all available patients, we did not perform a formal power analysis nor did we correct for multiple testing.

Subgroup analyses were performed for medical and surgical patients, those experiencing SAEs during the monitoring period and after the monitoring period, as well as SpO_2_ thresholds for patients without chronic obstructive pulmonary disease (COPD) and without severe obesity (body mass index < 40 kg/m^2^), along with subgroup analyses for those with COPD or body mass index ≥ 40 kg/m^2^ (i.e., accepting SpO_2_ ≥ 88% as normal).

Tables and figures present optimised thresholds, defined as those with the highest AUROC point estimates and a cut-off between 1 and 180 min, along with the AUROC, the corresponding 95% CI, the optimal cut-off, as well as the corresponding sensitivity and specificity. The optimised thresholds for the specific secondary outcomes with an AUROC point estimate >0.70 are also presented in tables with similar measures. Requirements for the analyses are listed in Supplemental Table S2. The prognostic ability of the identified thresholds was estimated using the same dataset from which the thresholds were derived. As this was an exploratory study, external validation is needed. All statistical analyses were performed using the statistical software R version 4.3.2 (R Core Team, Vienna, Austria).

## 3. Results

**Figure 2:**
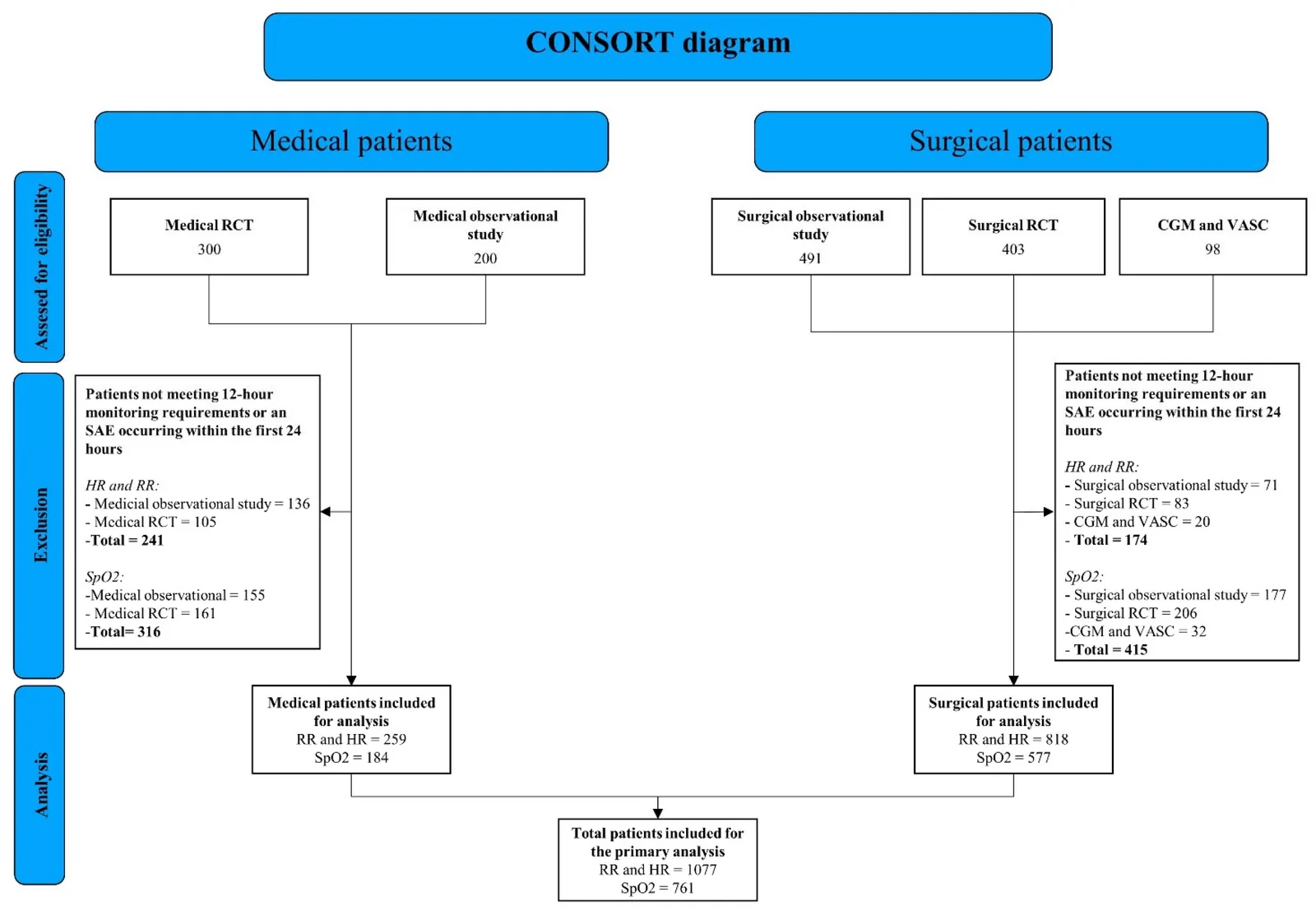
CONSORT diagram of patient inclusion and data analysis. Legend: RCT: randomised controlled trial; CGM: continuous glucose monitoring; VASC: vascular surgery; SAE: serious adverse event; HR: heart rate; RR: respiratory rate; SpO2: peripheral oxygen saturation.

A total of 1492 patients were assessed for eligibility. The primary analysis of HR and RR included 1077 patients, while 415 patients were excluded, as the requirement for duration of recording was not met for these variables. For SpO_2_, a total of 761 patients were included in the primary analysis, while 731 were excluded (Figure 2). We included 818 surgical patients and 259 medical patients; most patients were male (61%), median age was 70 years, 62% had previous cancer, and 21% had COPD (Table 1). The median full monitoring time was 46.9 h of valid HR/RR measurements and 36.8 h of valid SpO_2_ measurements. Median HR was 78 bpm (IQR 68–89), median RR was 16 brpm (IQR 13–19), and median SpO_2_ was 93% (IQR 91–95). In total, 30% of patients experienced one or more SAEs within 30 days. The types and frequencies of SAEs are detailed in Supplemental Table S3.

### 3.1. Primary Analysis

**Figure 3:**
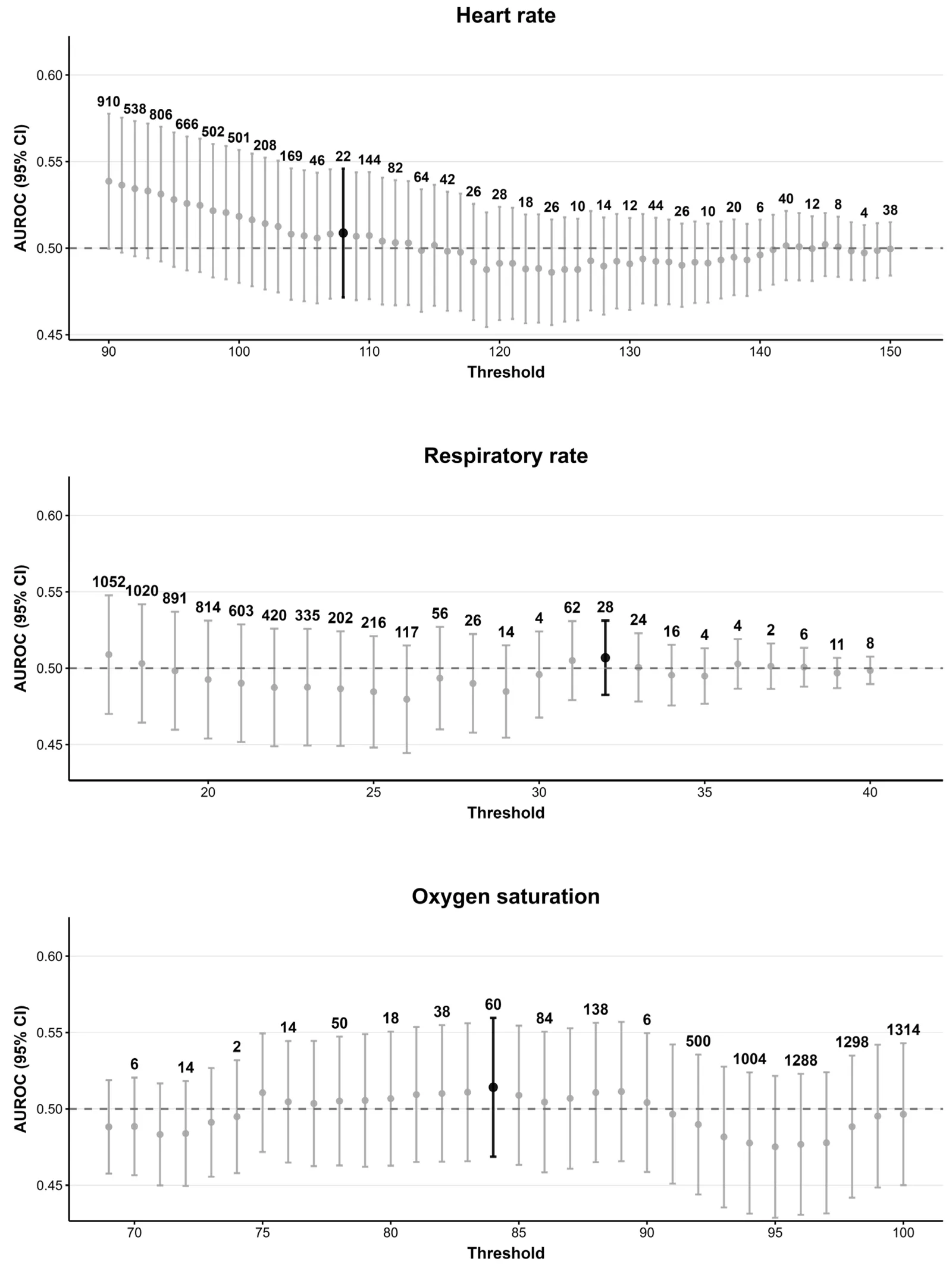
Prognostic ability of optimised thresholds for any SAE when comparing the last 24 h before an SAE with the last 24 h of monitoring in patients without SAE. Legend: AUROC: area under receiver operating characteristic curve; 95% CI: 95% confidence interval. The black-marked threshold represents the optimised threshold, defined as the highest AUROC with a cut-off between 1 and 180 min. This criterion was applied to emphasise thresholds with potential clinical relevance as alarms, whereas thresholds requiring prolonged deviations outside normal vital sign values were considered less applicable, as they more likely reflect general frailty rather than acute physiological deviation. Numbers above thresholds indicate the optimal cut-off in minutes.

In total, 70 different thresholds for duration below SpO_2_ thresholds, 130 thresholds for duration above HR, and 36 thresholds for duration above RR were assessed for prognostic ability (Supplemental Table S2). When comparing the last 24 h before an SAE with the last 24 h of monitoring in those without an SAE, all traditional NEWS-based thresholds had AUROC values no better or worse than chance, including HR > 130 bpm (AUROC 0.49, 95% CI 0.46–0.52), HR < 41 bpm (AUROC 0.50, 95% CI 0.48–0.51), RR > 24 brpm (AUROC 0.49, 95% CI 0.45–0.52), RR < 9 brpm (AUROC 0.47, 95% CI 0.43–0.50), and SpO_2_ < 92% (AUROC 0.49, 95% CI 0.44–0.54). All optimised thresholds for any SAE had low prognostic ability (Figure 3). Accumulated duration of HR > 108 bpm for ≥22 min, RR > 32 brpm for ≥28 min, and SpO_2_ < 84% for ≥60 min all had the highest AUROC point estimates of 0.51 and 95% CI 0.47–0.55, 0.48–0.53, and 0.47–0.56, respectively. In subgroup analyses, all thresholds had low prognostic ability, with HR > 91 bpm for ≥56 min in patients experiencing SAEs during monitoring demonstrating the highest AUROC of 0.65 (95% CI 0.56–0.73) (Table 2).

A total of 6456 thresholds were evaluated for their prognostic ability for specific SAE groups, and 84 optimised thresholds were identified for each combination of vital signs, SAE group, and population. Among these, 18 thresholds (21%) had AUROC point estimates > 0.70. SpO_2_ < 75% for ≥2 min and HR > 104 bpm for ≥36 min had the highest AUROC point estimates for respiratory SAEs during monitoring with 0.82 (95% CI 0.61–1.00) and 0.82 (95% CI 0.74–0.90), respectively. For all-cause mortality in both medical and surgical patients, RR > 23 brpm for ≥44 min and HR > 95 bpm for ≥165 min had AUROC estimates of 0.71 (95% CI 0.60–0.82) and 0.70 (95% CI 0.58–0.82), respectively, while SpO_2_ < 77% for ≥2 min in the medical subgroup had an AUROC of 0.70 (95% CI 0.53–0.87) (Table 3).

### 3.2. Secondary Analysis

Analyses of the last 24 h before any SAE compared with the first 24 h of monitoring in patients without an SAE had low prognostic ability (Supplemental Figure S2), with AUROC close to 0.50 for all vital sign thresholds (Supplemental Table S4). For specific SAE groups, 16 of 84 optimised thresholds (19%) reached AUROC > 0.70 (Supplemental Table S5).

### 3.3. Tertiary Analysis

Analyses of the last 12 h of measurements before any SAE compared to the period measured 12–24 h prior to the SAE had low prognostic ability (Supplemental Figure S3), with AUROC close to 0.50 for all vital sign thresholds (Supplemental Table S6). For specific SAE groups, seven of 84 optimised thresholds (8.3%) reached AUROC > 0.70 (Supplemental Table S7).

## 4. Discussion

The accumulated duration above or below thresholds for each vital sign had overall low prognostic ability for any SAE and no individual vital sign demonstrated superior prognostic ability. SpO_2_ showed the strongest prognostic ability for specific SAEs, with 26 optimised thresholds having moderate prognostic ability across analyses, compared to 16 optimised thresholds for HR and 13 optimised thresholds for RR. For the primary analysis, SpO_2_ < 75% for ≥2 min achieved the highest AUROC of 0.82 for respiratory SAE during monitoring. Optimised thresholds had overall low prognostic ability for any SAE but had higher point estimates of AUROC than traditional thresholds. NEWS-based thresholds often performed worse than chance when applied directly to CVSM, and a 60 min cut-off resulted in highly unbalanced sensitivity and specificity, limiting clinical applicability. The optimised thresholds demonstrated only marginally improved prognostic performance compared with traditional NEWS-based thresholds, and the clinical relevance of this modest improvement is uncertain and requires external validation.

Accumulated duration outside predefined traditional NEWS-based thresholds as well as episodes of tachycardia, tachypnoea, and desaturation in major abdominal surgery patients were previously assessed, and no significant association between vital sign abnormalities and subsequent SAEs were identified, specifically with NEWS-based thresholds of HR (>110 bpm for ≥60 min and >130 bpm for ≥30 min), RR (>24 brpm for ≥5 min and >30 brpm for ≥1 min), and SpO_2_ (<92% for ≥60 min, <88% for ≥10 min, <85% for ≥5 min, <80% for ≥1 min) [9]. Vital sign deviations were also assessed in patients admitted for acute exacerbation of chronic obstructive pulmonary disease (AECOPD) using similar thresholds. While abnormalities were substantial in AECOPD patients, no significant differences were observed between those with and without an SAE [8]. Our analyses confirmed this absence of significant association with the traditional thresholds [8,9]. Contrary to our hypothesis, the optimised thresholds were closer to normal while the optimal duration outside these thresholds varied substantially but was generally longer.

Thresholds for SAE during monitoring consistently had the highest AUROC estimates, which is expected as the exposure period directly precedes the event, making changes more imminent. Harriet et al. similarly found that CVSM summary measures closer to respiratory insufficiency were more strongly associated with the outcome than measurements further from it [23]. Thresholds for specific SAE groups generally yielded higher AUROC estimates, potentially reflecting distinct physiological changes preceding different SAEs. However, many of the highest AUROC estimates were based on few events, particularly for cardiovascular, respiratory, and neurologic SAEs, as well as SAEs occurring during monitoring. The limited number of SAEs may have affected AUROC estimates and limit the robustness of these findings [24]. Assessing thresholds across different exposure periods, specifically comparing the 24 h prior to an SAE with the first and final 24 h in patients without an SAE, demonstrated similar prognostic ability and threshold values. This indicates that varying the 24 h exposure periods in patients without SAEs had a minimal impact on prognostic ability. Comparing the last 12 h before an SAE to the preceding 12–24 h had the overall lowest prognostic ability, with the fewest thresholds reaching moderate prognostic ability, and the limited number of SAEs reduced statistical power. This study focused on optimised thresholds with durations under 180 min. Although some thresholds with higher AUROC were identified outside the reported ranges, they were not considered optimised, as prolonged deviations outside normal vital sign values (e.g., HR > 85) more likely reflect baseline deviation and frailty rather than acute pathology that can be intervened on. While CVSM captures physiological variability related to activity, these fluctuations are unlikely to explain the severity and duration of vital sign deviations that exceed optimised threshold limits. In a minority of patients, CVSM data were available to clinical staff as part of a clinical trial and may have influenced clinical management and some clinical outcomes [25], although this interventional change in vital signs likely is small in this study.

Certain thresholds had moderate prognostic ability for specific SAE groups when comparing the last 24 h before an SAE with the last 24 h of measurements in those without an SAE. RR > 23 brpm for ≥44 min and HR > 95 bpm for ≥165 min in all patients, as well as SpO_2_ < 77% for ≥2 min in the medical subgroup had moderate prognostic ability for 30-day mortality. This indicates that vital sign deviation measured over longer periods with CVSM may predict all-cause mortality beyond 24 h, an aspect not covered by NEWS, designed and validated for 24-h mortality prediction [26]. Ljunggren et al. associated single measurements of RR > 30 with significantly increased odds of 30-day mortality [27]. Our optimal threshold of RR > 23 for ≥44 min suggests that a lower threshold with prolonged deviation may enhance prognostic ability in CVSM.

Optimised CVSM thresholds had prognostic ability for respiratory and infectious SAE during monitoring. The optimal thresholds for RR (>32 brpm for ≥2 min) and SpO_2_ (<75% for ≥2 min) were outside traditional NEWS-based thresholds, possibly explaining the previously reported limited prognostic ability [8,9]. An optimal duration of 2 min suggests that even short periods of severe deviations may indicate respiratory distress and imminent respiratory SAE. Jacobi et al. described tachycardia and tachypnoea as components of the systemic inflammatory response syndrome in sepsis [28]. Despite our study also including less severe outcomes, the observed physiological changes are clinically plausible, as respiratory SAEs (e.g., pneumonia, atelectasis) and infectious SAEs (e.g., sepsis, urinary tract infections) were likely preceded by a degree of systemic inflammatory response.

When comparing the last 24 h before an SAE with the last 24 h of measurements in those without an SAE, SpO_2_ < 85% for ≥150 min in medical patients and SpO_2_ < 79% for ≥6 min in COPD patients had moderate prognostic ability for cardiovascular SAEs. When comparing the last 12 h before an SAE to the preceding 12–24 h, SpO_2_ < 83% for ≥2 min in the final 12 h also had moderate prognostic ability for cardiovascular SAEs, suggesting SpO_2_ changes precede these events. Loft et al. evaluated deviations from predefined vital sign thresholds prior to myocardial injury and described a significant association between accumulated durations of severe hypoxemia (SpO_2_ < 88%, SpO_2_ < 85% and SpO_2_ < 80%), tachycardia (HR > 110 bpm and HR > 130 bpm), and tachypnoea (RR > 24 brpm and RR > 30 brpm), and subsequent myocardial injury [29]. While our study did not identify similar associations for tachycardia and tachypnoea over 24h monitoring periods, possibly due to not analysing patients with myocardial injury as a separate subgroup, we found that HR > 102 for ≥2 min in the 12 h before cardiovascular SAEs had moderate prognostic ability, suggesting tachycardia as a potential early indicator of such events.

Anusic et al. identified CVSM thresholds in general surgical wards that reduced alarm frequency to a manageable level and were perceived relevant by nurses, but sensitivity and specificity for SAEs were not assessed [30]. The goal should be generating alarms that enable early and accurate detection of clinical deterioration to potentially prevent SAEs. Anderson et al. suggested simple interventions to address alarm fatigue, including ongoing evaluation of each patient’s need for CVSM, demonstrating a 17% reduction in alarms [31]. Even larger alert reduction of >80% are seen when thresholds are combined with temporal criteria, without loss of sensitivity to SAEs [32]. The identified threshold and duration combinations in this study maximised the combined sensitivity and specificity. The optimal threshold depends on both the type of SAE and the clinical setting. For some SAEs, higher sensitivity may be preferred despite an increased alarm burden. Current evidence indicate that CVSM reduces workload of the clinical staff and is considered safe with minimal device-related adverse effects [33,34].

This study included a large sample with a substantial quantity of continuous data of both medical and surgical patients. Previous studies have focused on fixed predefined vital sign thresholds, which provide standardisation but do not optimise prognostic ability [8,9]. Systematically quantifying multiple thresholds enabled a more precise assessment of their effectiveness in detecting patient deterioration and identified those with the highest prognostic ability.

First, given the large number of evaluated thresholds across different vital signs, patient subgroups, and SAE types, the risk of type I errors and false positive findings is increased. Consequently, some of the identified thresholds with high AUROC values may have occurred by chance rather than representing true associations. Thus, the identified thresholds require validation in independent cohorts before clinical implementation. Second, the population was included in prospective studies with specific inclusion and exclusion criteria, which to some extent limit the generalisability to the younger population and those not able to consent. Third, the prognostic ability was assessed for thresholds of individual vital signs, but combining multiple parameters could improve prognostic performance further, while also complicating clinical interpretation and application.

Our study found overall low prognostic ability for the broad definition of any SAEs, but certain vital sign thresholds had moderate prognostic ability for specific SAE groups. Despite heterogeneity in SAE types even within SAE groups, grouping was necessary to ensure thresholds remained broadly applicable, as monitoring systems must function across diverse patient populations and SAE types to be clinically relevant. The heterogeneity of the composite endpoint may have reduced prognostic performance, as individual SAE types are preceded by different, or potentially no, vital sign abnormalities. Larger datasets are needed to evaluate the prognostic ability of CVSM for each individual type of SAE. The predefined 24 h window limits real-time SAE prediction, as the true exposure period is unknown. Alarms based on accumulated duration can be directly implemented, as the monitoring system can count measurements and trigger an alarm once the cut-off is reached. However, the optimised thresholds identified in the present study are exploratory and require external validation before their clinical relevance and utility as real-time alarms can be established. Real-time analysis may allow faster intervention but require more data, specifically from patients experiencing SAEs during monitoring.

## 5. Conclusions

The accumulated duration outside thresholds for individual vital signs demonstrated overall low prognostic ability for any SAE. Optimised thresholds had higher point estimates of AUROC than traditional thresholds, but 95% CIs overlapped substantially, indicating considerable statistical uncertainty. The clinical impact of these differences requires further validation. Multiple thresholds had moderate prognostic ability for specific types of SAEs. These novel threshold criteria must be prospectively tested for their ability to identify SAEs.

## Figures and Tables

**Table 1:** Baseline characteristics of patients in the primary analysis of heart rate and respiratory rate.

	No SAE Within 30 Days n = 751	SAE Within 30 Days n = 326
Sex Female, n (%)	304 (41)	118 (37)
Age, median (IQR)	70 (64, 76)	71 (66, 75)
Patient population Surgical, n (%)	554 (74)	264 (81)
Medical, n (%)	197 (26)	62 (19)
Reason for admission, n (%)		
Pancreatic resection	99 (14)	90 (28)
Oesophagus resection	73 (10)	36 (11)
Ventricle resection	21 (2.9)	7 (2.2)
Small intestine or colon resection	142 (19)	29 (9.1)
Rectum resection	32 (4.4)	19 (6.0)
Other major surgical procedures	172 (23)	76 (24)
Sepsis	11 (1.5)	4 (1.3)
Dyspnoea	93 (13)	27 (8.5)
Pneumonia	43 (5.8)	15 (4.7)
Heart failure	1 (0.1)	1 (0.3)
AECOPD	49 (6.7)	15 (4.7)
Smoking status, n (%)		
Never	204 (27)	79 (24)
Former	390 (52)	189 (59)
Current	150 (20)	55 (17)
BMI, median (IQR)	25 (23, 29)	25 (22, 29)
Charlson comorbidity index, n (%)		
0	32 (4.3)	3 (0.9)
1–2	66 (8.9)	16 (5.0)
3–4	262 (35)	143 (31)
5–6	266 (36)	143 (44)
7–8	65 (8.8)	43 (13)
9+	50 (6.7)	17 (5.3)
Comorbidities, n (%)		
Diabetes mellitus	131 (18)	72 (22)
Chronic obstructive pulmonary disease	155 (21)	73 (22)
Other chronic pulmonary disease	42 (6.5)	16 (4.9)
Myocardial infarction	24 (3.2)	9 (2.8)
Cerebrovascular disease	57 (7.7)	21 (6.5)
Cancer	451 (61)	215 (67)
BMI ≥ 40 kg/m^2^	7 (0.9)	4 (1.2)
Baseline vital signs, median (IQR)		
Heart rate (bpm)	79 (69, 91)	79 (68, 91)
Respiratory rate (brpm)	16 (13, 18)	16 (13, 19)
Systolic blood pressure (mmHg)	137 (124, 152)	134 (122, 150)
Diastolic blood pressure (mmHg)	76 (68, 86)	77 (68, 84)
Peripheral oxygen saturation (%)	97 (96, 99)	98 (96, 99)

Legends: SAE: serious adverse event; n: number; IQR: interquartile range; AECOPD: acute exacerbation of chronic obstructive pulmonary disease; BMI: body mass index; bpm: beats per minute; brpm: breaths per minute; mmHg: millimetres of mercury.

**Table 2:** Prognostic ability of traditional NEWS-based thresholds and optimised thresholds for any SAE.

Threshold	Population	AUC (95%CI)	Cut-Off	Sensitivity	Specificity	Avg. Time Outside Threshold (SAE)	Avg. Time Outside Threshold (No SAE)
Traditional NEWS-based thresholds							
HR > 130	All patients	0.49 (0.46–0.52)	60	0.05	0.96	12	7.4
RR > 24	All patients	0.49 (0.45–0.52)	60	0.20	0.80	76	54
RR < 9	All patients	0.47 (0.43–0.50)	60	0.04	0.96	12	12
SpO2 < 92	All patients	0.49 (0.44–0.54)	60	0.81	0.17	370	376
HR < 41	All patients	0.50 (0.48–0.51)	5	0.02	0.98	0.4	0.9
Optimised thresholds							
HR > 104	All patients	0.51 (0.47–0.55)	169	0.20	0.85	123	84
HR > 105	All patients	0.51 (0.47–0.55)	50	0.34	0.72	112	77
HR > 107	All patients	0.51 (0.47–0.55)	32	0.35	0.70	93	63
HR > 108	All patients	0.51 (0.47–0.55)	22	0.37	0.68	84	58
HR > 95	Surgical subgroup	0.54 (0.50–0.58)	168	0.32	0.77	212	137
HR > 107	Medical subgroup	0.56 (0.48–0.65)	46	0.56	0.60	185	126
HR > 108	Medical subgroup	0.56 (0.48–0.65)	158	0.35	0.81	168	114
HR > 91	SAE during monitoring	0.65 (0.56–0.73)	56	0.86	0.39	455	269
HR > 142	SAE after monitoring	0.50 (0.48–0.52)	80	0.02	0.99	6.2	2.7
HR > 143	SAE after monitoring	0.50 (0.48–0.52)	60	0.02	0.99	5.8	2.5
HR > 144	SAE after monitoring	0.50 (0.48–0.52)	12	0.04	0.97	5.4	2.3
HR > 145	SAE after monitoring	0.50 (0.48–0.52)	36	0.03	0.98	5.0	2.1
HR > 146	SAE after monitoring	0.50 (0.48–0.52)	13	0.04	0.97	4.6	1.9
RR > 32	All patients	0.51 (0.48–0.53)	28	0.04	0.98	3.6	3.1
RR > 20	Surgical subgroup	0.52 (0.48–0.56)	165	0.30	0.77	164	120
RR > 30	Medical subgroup	0.57 (0.49–0.65)	4	0.45	0.70	33	15
RR > 30	SAE during monitoring	0.57 (0.49–0.65)	2	0.33	0.85	9.6	6.3
RR > 36	SAE after monitoring	0.50 (0.48–0.52)	4	0.03	0.98	0.6	0.9
RR > 38	SAE after monitoring	0.50 (0.49–0.51)	6	0.01	0.99	0.3	0.6
RR > 40	SAE after monitoring	0.50 (0.49–0.51)	8	0.01	0.99	0.1	0.4
SpO2 < 83	All patients	0.51 (0.47–0.56)	46	0.14	0.90	25	20
SpO2 < 84	All patients	0.51 (0.47–0.56)	60	0.16	0.89	35	27
SpO2 < 88	All patients	0.51 (0.47–0.56)	138	0.25	0.79	112	101
SpO2 < 89	All patients	0.51 (0.47–0.56)	138	0.36	0.69	153	142
SpO2 < 89	Surgical subgroup	0.54 (0.49–0.59)	146	0.27	0.82	129	89
SpO2 < 90	Surgical subgroup	0.54 (0.49–0.59)	176	0.33	0.75	176	131
SpO2 < 81	Medical subgroup	0.59 (0.49–0.69)	20	0.41	0.74	26	20
SpO2 < 82	Medical subgroup	0.59 (0.49–0.69)	27	0.41	0.76	37	28
SpO2 < 84	Medical subgroup	0.59 (0.49–0.69)	10	0.79	0.40	69	55
SpO2 < 84	SAE during monitoring	0.58 (0.46–0.69)	8	0.63	0.56	66	27
SpO2 < 75	SAE after monitoring	0.51 (0.47–0.55)	2	0.23	0.79	2.2	2.1
SpO2 < 88	SAE after monitoring	0.51 (0.46–0.56)	138	0.26	0.79	106	101
SpO2 < 90	SAE after monitoring	0.51 (0.46–0.56)	6	0.93	0.12	206	199
SpO2 < 84	COPD	0.55 (0.46–0.64)	9	0.80	0.34	72	60
SpO2 < 88	Without COPD and BMI < 40	0.51 (0.46–0.56)	137	0.19	0.88	81	67

Legend: Analysis conducted by comparing the last 24 h before the event with the last 24 h of monitoring in those without serious adverse events. HR: heart rate per minute; RR: respiratory rate per minute; SpO2: peripheral oxygen saturation in %; SAE: serious adverse event; NEWS: National Early Warning Score; AUC: area under the curve; 95% CI: 95% confidence interval; COPD: chronic obstructive pulmonary disease; BMI: body mass index. Only thresholds with a cut-off < 180 are presented. Number of patients in HR and RR analyses (no SAE/SAE): Total = 1077 (751/326), surgical subgroup = 818 (554/264), medical subgroup = 259 (197/62), SAE during monitoring = 794 (751/43), SAE after monitoring = 1034 (751/283). Number of patients in SpO_2_ analysis (no SAE/SAE): total = 761 (540/221), surgical subgroup = 577 (395/182), medical subgroup = 184 (145/39), SAE during monitoring = 570 (540/30), SAE after monitoring = 731 (540/191), COPD = 167 (118/49), without COPD and BMI<40 = 590 (419/171).

**Table 3:** Prognostic ability of optimised thresholds for specific SAE groups.

Threshold	SAE Group	Population	n SAE	AUC (95% CI)	Cut-Off	Sensitivity	Specificity	Avg. Time Outside Threshold (SAE)	Avg. Time Outside Threshold (No SAE)
HR > 104	Respiratory SAE	SAE during monitoring	8	0.82 (0.74–0.90)	36	1.00	0.64	199	84
HR > 105	Respiratory SAE	SAE during monitoring	8	0.82 (0.74–0.90)	30	1.00	0.64	175	77
HR > 102	Respiratory SAE	SAE during monitoring	8	0.82 (0.73–0.91)	40	1.00	0.60	247	102
HR > 103	Respiratory SAE	SAE during monitoring	8	0.82 (0.73–0.91)	40	1.00	0.63	221	93
SpO2 < 75	Respiratory SAE	SAE during monitoring	6	0.82 (0.61–1.00)	2	0.83	0.79	19	2.1
SpO2 < 85	Cardiovascular SAE	Medical subgroup	4	0.81 (0.61–1.00)	150	0.75	0.86	174	76
SpO2 < 79	Cardiovascular SAE	COPD	5	0.80 (0.66–0.94)	6	1.00	0.58	28	12
HR > 118	Neurologic SAE	SAE during monitoring	2	0.79 (0.61–0.98)	4	1.00	0.69	22	22
HR > 119	Neurologic SAE	SAE during monitoring	2	0.79 (0.60–0.99)	2	1.00	0.69	18	20
SpO2 < 74	All-cause mortality	Without COPD and BMI < 40	8	0.79 (0.60–0.98)	2	0.62	0.88	6.1	1.0
SpO2 < 75	All-cause mortality	Without COPD and BMI < 40	8	0.79 (0.60–0.98)	2	0.75	0.84	7.6	1.3
HR > 120	Neurologic SAE	SAE during monitoring	2	0.79 (0.59–1.00)	2	1.00	0.68	17	18
SpO2 < 76	All-cause mortality	Without COPD and BMI < 40	8	0.79 (0.59–0.99)	2	0.75	0.80	10	1.6
RR > 34	Other SAE	Medical subgroup	7	0.78 (0.57–0.99)	2	0.71	0.83	21	2.9
SpO2 < 89	Neurologic SAE	Without COPD and BMI < 40	2	0.77 (0.54–0.99)	82	1.00	0.65	151	95
SpO2 < 84	Neurologic SAE	Surgical subgroup	3	0.76 (0.69–0.83)	10	1.00	0.70	14	17
SpO2 < 84	Neurologic SAE	All patients	4	0.75 (0.59–0.92)	10	1.00	0.62	108	27
SpO2 < 84	Neurologic SAE	SAE after monitoring	4	0.75 (0.59–0.92)	10	1.00	0.62	108	27
HR > 89	Infectious SAE	SAE during monitoring	7	0.74 (0.60–0.87)	146	1.00	0.49	602	314
RR > 32	Respiratory SAE	SAE during monitoring	8	0.74 (0.55–0.92)	2	0.62	0.88	7.6	3.1
RR > 22	All-cause mortality	All patients	25	0.71 (0.60–0.82)	108	0.64	0.75	284	105
RR > 23	All-cause mortality	All patients	25	0.71 (0.60–0.82)	44	0.68	0.71	225	75
HR > 95	All-cause mortality	All patients	25	0.70 (0.58–0.82)	165	0.68	0.68	463	192
SpO2 < 77	All-cause mortality	Medical subgroup	8	0.70 (0.53–0.87)	2	0.88	0.59	13	6.0

Legend: Analysis conducted by comparing the last 24 h before the event with the last 24 h of monitoring in those without serious adverse events. HR: heart rate per minute; RR: respiratory rate per minute; SpO2: peripheral oxygen saturation in %; SAE: serious adverse event; AUC: area under the curve; 95% CI: 95% confidence interval; COPD: chronic obstructive pulmonary disease; BMI: body mass index; n: number. Only thresholds with a cut-off < 180 are presented. Number of patients without SAEs in HR and RR analyses: total = 751, surgical = 554, medical = 197. Number of patients without SAEs in SpO2 analysis: total = 540, surgical = 395, medical = 145, COPD = 118, without COPD and BMI < 40 = 419.

## Data Availability

The data are not publicly available due to the sensitive nature of the study and because participants did not provide consent for public data sharing.

## Supplementary Materials

The following supporting information can be downloaded at: https://www.mdpi.com/article/10.3390/s26175399/s1, Figure S1: Data collection setup; Figure S2: Prognostic ability of optimised thresholds for any SAE when comparing the last 24 h before an SAE with the first 24 h of monitoring in patients without SAEs; Figure S3: Prognostic ability of optimised thresholds for any SAE when comparing measurements from the last 12 h before an SAE to those measured 12–24 h prior; Table S1: Inclusion and exclusion criteria for all studies; Table S2: Criteria for analysis and inclusion of thresholds in Tables; Table S3: Overview of serious adverse events in analysis of heart rate and respiratory rate; Table S4: Prognostic ability of optimised thresholds for any SAE when comparing the last 24 h before an SAE with the first 24 h of monitoring in patients without SAEs; Table S5: Prognostic ability of optimised thresholds for SAE groups when comparing the last 24 h before an SAE with the first 24 h of monitoring in patients without SAEs; Table S6: Prognostic ability of optimised thresholds for any SAE when comparing measurements from the last 12 h before an SAE to those measured 12–24 h prior; Table S7: Prognostic ability of optimal thresholds for SAE groups when comparing measurements from the last 12 h before an SAE to those measured 12–24 h prior.

## Abbreviations

The following abbreviations are used in this manuscript:

NEWS	National Early Warning Score
CVSM	Continuous vital sign monitoring
HR	Heart rate
SpO_2_	Peripheral oxygen saturation
RR	Respiratory rate
SAE	Serious adverse event
ECG	Electrocardiogram
brpm	Breaths per minute
bpm	Beats per minute
AUROC	Area under receiver operating characteristics curve
CI	Confidence interval
COPD	Chronic obstructive pulmonary disease
AECOPD	Acute exacerbation of chronic obstructive pulmonary disease
