# Supplementary material for: Prognostic Value of Traditional and Optimised Vital Sign Thresholds for Risk of Serious Adverse Events in Continuously Monitored Hospitalised Patients
Source: Sensors (Basel). 2026 Aug 26;26(17):5399. doi: 10.3390/s26175399 (PMC13567922; doi:10.3390/s26175399)
Supplement: Supplementary file 1 [file sensors-26-05399-s001.zip › sensors-4369286-supplementary.pdf]

sensors

MDPI

Supplementary Material—Prognostic value of traditional and optimised vital sign thresholds for risk of serious adverse events in continuously monitored hospitalised patients

Table of contents

Figure S1: Data collection setup 2

Figure S2: Prognostic ability of optimised thresholds for any SAE when comparing the last 24 hours before an SAE with the first 24 hours of monitoring in patients without SAEs........ 3

Figure S3: Prognostic ability of optimised thresholds for any SAE when comparing measurements from the last 12 hours before an SAE to those measured 12–24 hours prior........ 4

Table S1: Inclusion and exclusion criteria for all studies........ 5

Table S2: Criteria for analysis and inclusion of thresholds in Tables 6

Table S3: Overview of serious adverse events in analysis of heart rate and respiratory rate........ 7

Table S4: Prognostic ability of optimised thresholds for any SAE when comparing the last 24 hours before an SAE with the first 24 hours of monitoring in patients without SAEs........ 8

Table S5: Prognostic ability of optimised thresholds for SAE groups when comparing the last 24 hours before an SAE with the first 24 hours of monitoring in patients without SAEs 9

Table S6: Prognostic ability of optimised thresholds for any SAE when comparing measurements from the last 12 hours before an SAE to those measured 12–24 hours prior........ 10

Table S7: Prognostic ability of optimal thresholds for SAE groups when comparing measurements from the last 12 hours before an SAE to those measured 12–24 hours prior........ 11

 https://doi.org/10.3390/s26175399

Figure S1: Data collection setup

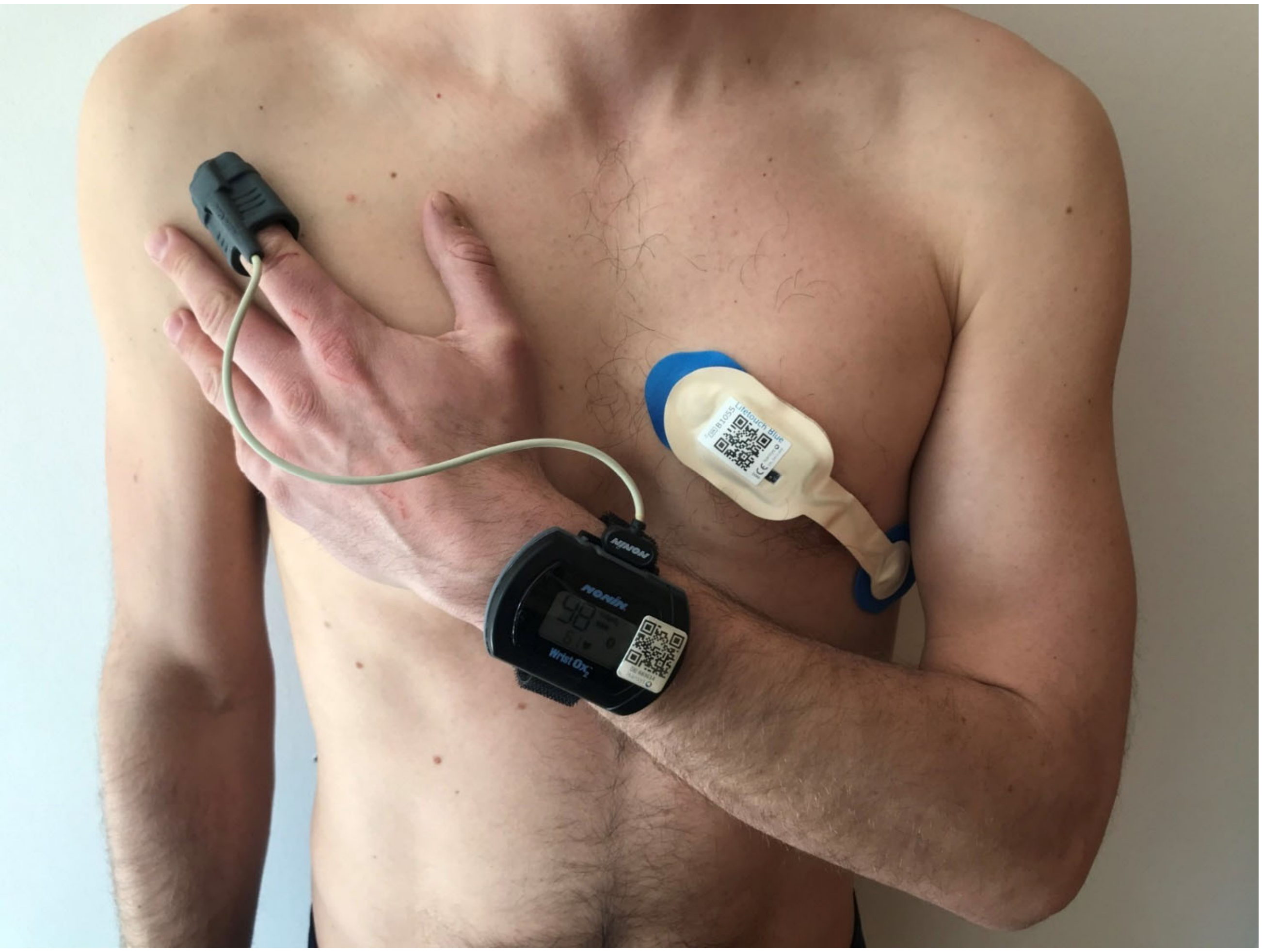

Legends: *Data were collected using the clinically validated CE- and FDA-approved Isansys Lifetouch (Isansys Lifecare, Abingdon, Oxfordshire, United Kingdom) and Nonin WristOx 3150 (Nonin Medical inc., Plymouth, MN, USA) [12,13].*

Isansys LifeTouch: Single-lead ECG patch placed on the left anterior chest using two electrodes 15 cm apart, measuring heart rate and ECG-derived respiratory rate.

Nonin WristOx 3150: Fingertip pulse oximeter measuring peripheral oxygen saturation.

Photo: Author's own photograph.

https://doi.org/10.3390/s26175399

Figure S2. Prognostic ability of optimised thresholds for any SAE when comparing the last 24 hours before an SAE with the first 24 hours of monitoring in patients without SAEs.

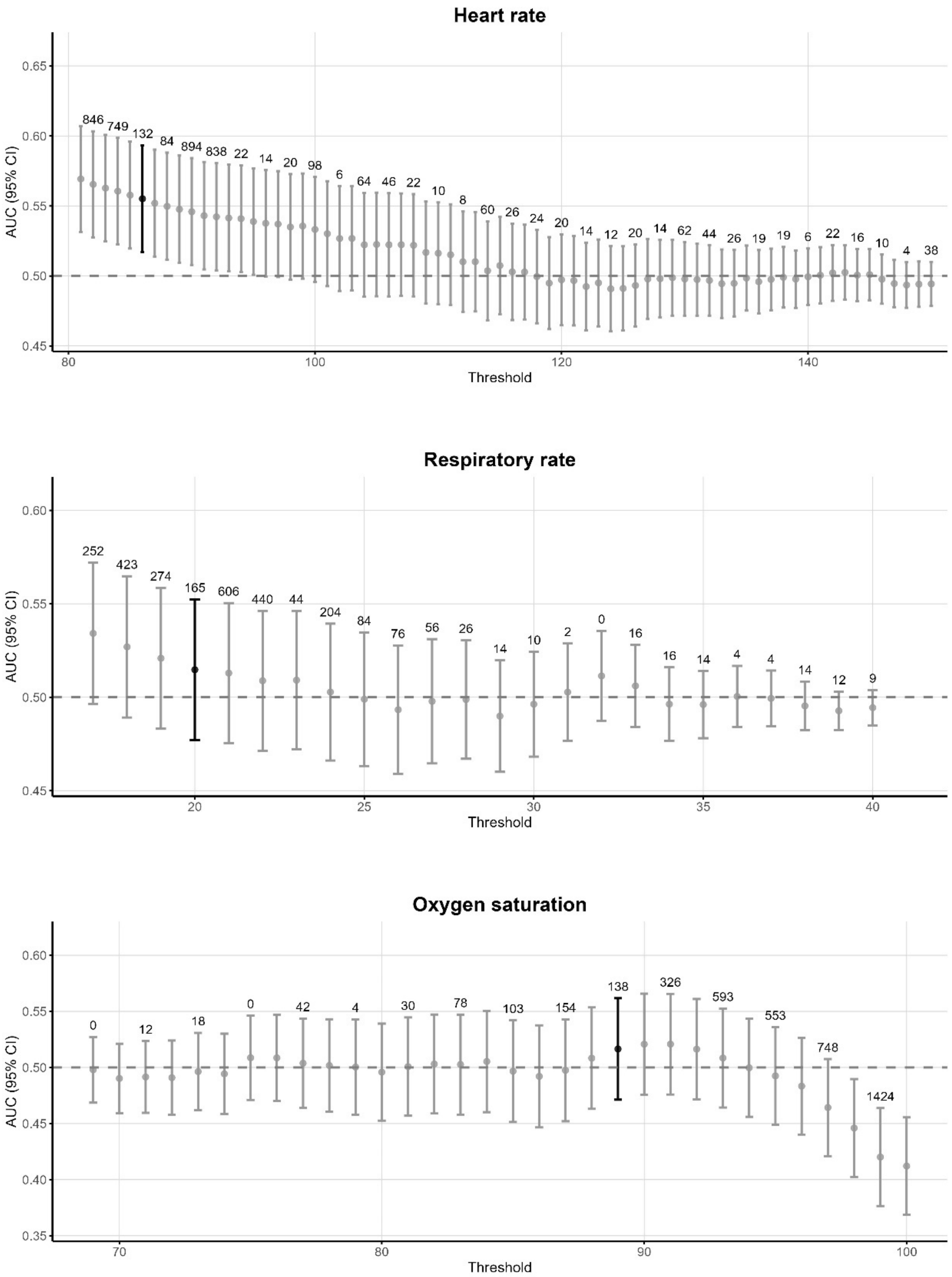

Legend: *AUC: area under the curve; 95% CI: 95% confidence interval.*

The black-marked threshold represents the optimised threshold, defined as the highest AUC with a cut-off between 1 and 180 minutes. This criterion was applied to emphasise thresholds with potential clinical relevance as alarms, whereas thresholds requiring prolonged deviations outside normal vital sign values were considered less applicable, as they more likely reflect general frailty rather than acute physiological deviation. Numbers above thresholds indicate the optimal cut-off in minutes

Figure S3: Prognostic ability of optimised thresholds for any SAE when comparing measurements from the last 12 hours before an SAE to those measured 12–24 hours prior

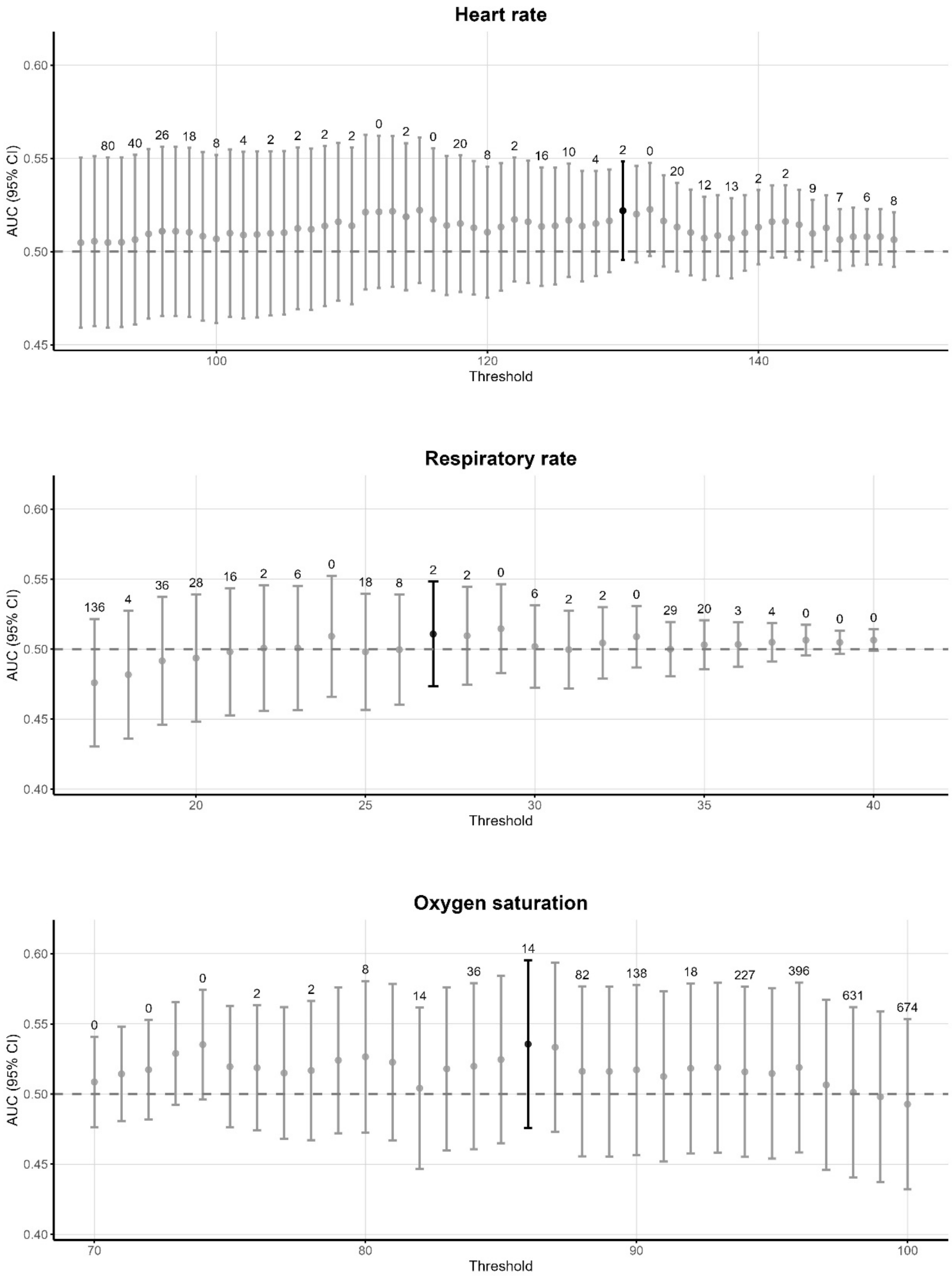

Legend: *AUC: area under the curve; 95% CI: 95% confidence interval.*

The black-marked threshold represents the optimised threshold, defined as the highest AUC with a cut-off between 1 and 180 minutes. This criterion was applied to emphasise thresholds with potential clinical relevance as alarms, whereas thresholds requiring prolonged deviations outside normal vital sign values were considered less applicable, as they more likely reflect general frailty rather than acute physiological deviation. Numbers above thresholds indicate the optimal cut-off in minutes

https://doi.org/10.3390/s26175399

Table S1: Inclusion and exclusion criteria for all studies

Study	Inclusion criteria	Exclusion criteria
Medical RCT<br>NCT04661748<br>H-20033246	• Randomisation possible within 12 hours<br>• Adult patients (≥18 years)<br>• At least one expected overnight stay<br>• High-risk medical admission*	• Inability to cooperate or give informed consent<br>• Allergy to plaster or silicone<br>• Admitted for palliative care<br>• Admission to telemetry unit<br>• Previously enrolled in a WARD-study<br>• A pacemaker or ICD device
Surgical RCT<br>NCT04640415<br>H-20034555	• Adult patients (≥50 years<br>• Major abdominal, orthopaedic, urologic, or vascular surgery<br>• Duration of surgery ≥2 hours<br>• At least two expected overnight stays<br>• Randomisation possible within 24 hours from ward arrival	• Inability to cooperate or give informed consent<br>• Allergy to plaster or silicone<br>• Impaired cognitive function or MMSE score < 24<br>• Admitted for palliative care<br>• Admission to telemetry unit<br>• Previously enrolled in a WARD-study<br>• A pacemaker or ICD device
Medical observational study<br>NCT03660501<br>H-18026653	• Adult patients (≥18 years)<br>• Admission for AECOPD<br>• Initiation of monitoring within 24 hours	• Inability to cooperate<br>• Allergy to plaster or silicone<br>• A pacemaker or ICD device
Surgical observational study<br>NCT03491137<br>H-17033535	• Adult patients (≥60 years)<br>• Elective major abdominal cancer surgery<br>• estimated incision time of ≥2 hours	• Inability to cooperate or give informed consent<br>• Allergy to plaster or silicone<br>• Impaired cognitive function or MMSE score < 24<br>• Admitted for palliative care<br>• Admission to telemetry unit<br>• A pacemaker or ICD device
Continuous glucose monitoring<br>NCT04473001<br>H-20002220	• Adult patients (≥18 years)<br>• Major abdominal, orthopaedic, or vascular surgery<br>• estimated incision time of ≥1 hour<br>• At least one expected overnight stay<br>• No DM, type 1, or type 2 DM	• Inability to cooperate or give informed consent<br>• Allergy to plaster or silicone<br>• Impaired cognitive function or MMSE score < 24<br>• Any treatment limitation<br>• Previous or scheduled pancreatectomy<br>• A pacemaker or ICD device
Vascular surgery<br>NCT04628858<br>H-19086583	• Adult patients (≥18 years)<br>• PAD patients undergoing open infraligamental revascularisation or bypass<br>• At least two expected overnight stays	• Inability to cooperate or give informed consent<br>• Allergy to plaster or silicone<br>• Impaired cognitive function<br>• >20 mmHg systolic BP difference between arms<br>• Previously enrolled in a WARD-study<br>• Any treatment limitation<br>• A pacemaker or ICD device

Legend: *RCT: randomised controlled trial; WARD: Wireless Assessment of Respiratory and circulatory Distress; ICD: implantable cardioverter defibrillator; MMSE: mini-mental state examination; AECOPD: acute exacerbation of chronic obstructive pulmonary disease; DM: diabetes mellitus; PAD: peripheral arterial*

disease; BP: blood pressure; mmHg: milimetres of mercury.

**High-risk medical admission includes discharge from ICU stay ≥24 hours or the following tentative diagnosis with at least two predefined vital sign deviations: pneumonia, dyspnea, acute coronary syndrome, new onset heart failure, or sepsis*

Reproduced from: N. Aagaard et al., Journal of Clinical Monitoring and Computing, 2024 [11], under the terms of the Creative Commons Attribution 4.0 International License (CC BY 4.0, https://creativecommons.org/licenses/by/4.0/).

Table S2: Criteria for analysis and inclusion of thresholds in the tables

Requirement	Description
For all analyses	
Patients	Adult patients (≥18 years) undergoing major non-cardiac surgery or admitted for acute medical disease
Monitoring time	Each included patient must have at least 50% valid measurements of either HR, RR or SpO_2 within the given exposure period
First SAE	Only vital sign measurements preceding the first SAE were included to avoid bias of vital sign deviation due to the first SAE and the medical interventions to correct it
Predictor variable	Accumulated duration above thresholds was used as the predictor variable for analysis of HR and RR, while accumulated duration below thresholds was the predictor variable for SpO2
Investigated thresholds	The thresholds investigated ranged from 20 to 150 for HR, 4 to 40 for RR, and 30% to 100% for SpO2
Cut-off	The threshold must have an optimal duration that is also considered clinically relevant, defined as being between 1 and 180 minutes. The optimal cut-offs were defined as the values of the predictor variable, specifically the duration outside the threshold, that maximised the sum of specificity and sensitivity. Traditional NEWS thresholds

	used an a priori 60-minute cut-off for HR >130 bpm, RR >24/<9 brpm, SpO_2 <92%, and 5-minute cut-off for HR <41 bpm
Decimals	Thresholds with an identical point-estimate of AUROC within the first two decimals are all presented
For analyses of secondary outcomes	
Prognostic ability	Area under receiver operating characteristics curve must be ≥0.70
Number of SAEs	A minimum of three occurrences of the specific SAE group is required for the threshold to be presented from the tertiary analysis

Legends: *SAE: serious adverse event; NEWS: National Early Warning Score; HR: heart rate; RR: respiratory rate;* SpO_2*: peripheral oxygen saturation; AUROC: area under receiver operating characteristic curve*

Table S3. Overview of serious adverse events in analysis of heart rate and respiratory rate.

SAE type	First SAE used for analysis<br>n	All SAEs within 30 days<br>n
Respiratory		
Atelectasis	3	7
Pleural effusion	22	41
Pneumonia	24	53
Pneumothorax	3	7
Respiratory failure	9	31
Cardiovascular		
Atrial flutter	3	4
Atrial fibrillation	11	18
Deep vein thrombosis	3	6
Heart failure	6	19
Myocardial infarction	0	1
Non-fatal cardiac arrest	0	2
Other supraventricular tachyarrhythmias	0	1
Pulmonary embolism	2	3
Second degree AV block	0	2
Third degree AV block	0	1
Myocardial injury	1	3
Ventricular tachycardia	2	2
Neurologic		
Delirium	7	12
Stroke	1	2
Syncope	1	3
Transient ischemic attack	0	2
Infectious		
Sepsis	7	38
Septic shock	0	12
Surgical site infection	59	109
Urinary tract infection	12	20

Other		
Bowel obstruction	28	45
Diabetic ketoacidosis	0	2
Pus, blood or fluid in wound or cavity	6	116
Fracture	1	1
Hypoglycaemia	0	2
Major bleeding	8	20
Opioid intoxication	1	5
Acute renal failure	2	11
Other SAE	79	102
All-cause mortality	NA	25*
**Total**	**301**	**728**

Legends: *SAE: serious adverse event; AV: atrio-ventricular; NA: not available; n: number.*

**All-cause mortality within 30 days was also used for analysis.*

Table S4. Prognostic ability of optimised thresholds for any SAE when comparing the last 24 hours before an SAE with the first 24 hours of monitoring in patients without SAEs.

Threshold	Population	AUC (95%CI)	Cut-off	Sensitivity	Specificity	Avg. time outside threshold (SAE)	Avg. time outside threshold (No SAE)
HR > 86	All patients	0.56 (0.52-0.59)	132	0.69	0.41	492	388
HR > 86	Surgical subgroup	0.59 (0.54-0.63)	132	0.66	0.50	429	275
HR > 108	Medical subgroup	0.53 (0.45-0.61)	160	0.35	0.78	168	144
HR > 86	SAE during monitoring	0.66 (0.58-0.74)	174	0.81	0.45	615	388
HR > 98	SAE after monitoring	0.52 (0.48-0.56)	176	0.29	0.76	200	155
HR > 99	SAE after monitoring	0.52 (0.48-0.56)	119	0.33	0.72	185	143
HR > 100	SAE after monitoring	0.52 (0.48-0.56)	98	0.34	0.72	169	132
RR > 20	All patients	0.51 (0.48-0.55)	165	0.37	0.68	233	205
RR > 20	Surgical subgroup	0.57 (0.52-0.61)	156	0.31	0.83	164	98
RR > 31	Medical subgroup	0.56 (0.49-0.64)	2	0.42	0.74	22	8.9
RR > 19	SAE during monitoring	0.59 (0.50-0.67)	76	0.74	0.43	391	269
RR > 20	SAE after monitoring	0.50 (0.47-0.54)	165	0.37	0.68	220	205
RR > 23	SAE after monitoring	0.50 (0.47-0.54)	44	0.30	0.73	95	84
RR > 32	SAE after monitoring	0.50 (0.48-0.53)	42	0.04	0.99	3.6	2.8
SpO2 < 89	All patients	0.52 (0.47-0.56)	138	0.36	0.71	153	133
SpO2 < 91	Surgical subgroup	0.56 (0.51-0.61)	142	0.51	0.60	240	174
SpO2 < 84	Medical subgroup	0.61 (0.51-0.70)	14	0.74	0.49	69	52
SpO2 < 84	SAE during monitoring	0.57 (0.46-0.69)	8	0.63	0.57	66	27
SpO2 < 88	SAE after monitoring	0.51 (0.46-0.56)	148	0.25	0.81	106	97
SpO2 < 84	COPD	0.58 (0.48-0.67)	10	0.80	0.40	72	57
SpO2 < 94	Without COPD and BMI<40	0.51 (0.46-0.56)	128	0.92	0.14	572	553

Legend*: HR: heart rate; RR: respiratory rate; SpO2: peripheral oxygen saturation in %; SAE: serious adverse event; AUC: area under the curve; 95% CI: 95% confidence interval; COPD: chronic obstructive pulmonary disease; BMI: body mass index. Only thresholds with a cut-off <180 are presented.*

Number of patients in HR and RR analyses (no SAE/SAE): total = 1100 (774/326), surgical subgroup = 832 (568/264), medical subgroup = 268 (206/62), SAE during monitoring = 817 (774/43), SAE after monitoring = 1057 (774/283)

Number of patients in SpO_2 *analyses (no SAE/SAE): total = 862 (641/221), surgical subgroup = 645 (463/182), medical subgroup = 217 (178/39), SAE during monitoring = 671 (641/30), SAE after monitoring = 832 (641/191), COPD = 190 (141/49), without COPD and BMI<40 = 668 (497/171)*

Table S5. Prognostic ability of optimised thresholds for SAE groups when comparing the last 24 hours before an SAE with the first 24 hours of monitoring in patients without SAEs.

Threshold	SAE group	Population	n SAE	AUC (95%CI)	Cut-off	Sensitivity	Specificity	Avg. time outside threshold (SAE)	Avg. time outside threshold (No SAE)
HR > 105	Respiratory SAE	SAE during monitoring	8	0.82 (0.75-0.89)	30	1.00	0.66	175	85
HR > 102	Respiratory SAE	SAE during monitoring	8	0.82 (0.74-0.90)	40	1.00	0.63	247	111
SpO2 < 86	Cardiovascular SAE	Medical subgroup	4	0.82 (0.67-0.97)	66	1.00	0.60	227	97
SpO2 < 85	Cardiovascular SAE	Medical subgroup	4	0.82 (0.64-1.00)	150	0.75	0.87	174	71
SpO2 < 75	Respiratory SAE	SAE during monitoring	6	0.81 (0.61-1.00)	2	0.83	0.78	19	2.6
SpO2 < 79	Cardiovascular SAE	COPD	5	0.80 (0.67-0.93)	6	1.00	0.62	28	15
HR > 118	Neurologic SAE	SAE during monitoring	2	0.80 (0.63-0.98)	4	1.00	0.71	22	24
SpO2 < 90	Neurologic SAE	Without COPD and BMI<40	2	0.79 (0.60-0.99)	136	1.00	0.69	217	127
SpO2 < 74	All-cause mortality	Without COPD and BMI<40	8	0.78 (0.60-0.97)	2	0.62	0.86	6.1	1.2
RR > 34	Other SAE	Medical subgroup	7	0.78 (0.58-0.98)	2	0.71	0.83	21	2.0
SpO2 < 84	Neurologic SAE	Surgical subgroup	3	0.76 (0.70-0.82)	10	1.00	0.70	14	18
SpO2 < 84	Neurologic SAE	All patients	4	0.76 (0.60-0.92)	10	1.00	0.63	108	27
SpO2 < 84	Neurologic SAE	SAE after monitoring	4	0.76 (0.60-0.92)	10	1.00	0.63	108	27
HR > 89	Infectious SAE	SAE during monitoring	7	0.74 (0.60-0.87)	146	1.00	0.49	602	314
RR > 32	Respiratory SAE	SAE during monitoring	8	0.74 (0.56-0.93)	2	0.62	0.89	7.6	2.8
RR > 25	All-cause mortality	Surgical subgroup	9	0.73 (0.55-0.90)	14	0.56	0.86	27	15
RR > 22	All-cause mortality	All patients	25	0.72 (0.61-0.82)	20	0.88	0.53	284	115
SpO2 < 77	All-cause mortality	Medical subgroup	8	0.70 (0.53-0.87)	2	0.88	0.59	13	7.2

Legend: *HR: heart rate; RR: respiratory rate; SpO2: peripheral oxygen saturation in %; SAE: serious adverse event; n: number; AUC: area under the curve; 95% CI: 95% confidence interval; COPD: chronic obstructive pulmonary disease; BMI: body mass index. Only thresholds with a cut-off <180 are presented.*

Number of patients without SAEs HR and RR: total = 774, surgical = 568, medical = 206
Number of patients without SAEs SpO2: total = 641, surgical = 463, medical = 178, COPD = 141, without COPD and BMI<40 = 497

https://doi.org/10.3390/s26175399

Table S6. Prognostic ability of optimised thresholds for any SAE when comparing measurements from the last 12 hours before an SAE to those measured 12–24 hours prior.

Threshold	Population	AUC (95%CI)	Cut-off	Sensitivity	Specific-ity	Avg. time out-side threshold (0-12 hours prior to SAE)	Avg. time out-side threshold (12-24 hours prior to SAE)
HR > 130	All patients	0.52 (0.50-0.55)	2	0.13	0.92	5.3	7.4
HR > 130	Surgical subgroup	0.53 (0.51-0.56)	2	0.13	0.94	4.3	1.3
HR > 113	Medical subgroup	0.48 (0.38-0.59)	2	0.61	0.46	34	76
HR > 130	SAE during monitoring	0.60 (0.51-0.68)	2	0.26	0.93	4.7	3.8
HR > 132	SAE during monitoring	0.60 (0.52-0.67)	2	0.24	0.95	3.6	3.3
RR > 27	All patients	0.51 (0.47-0.55)	2	0.27	0.77	12	17
RR > 27	Surgical subgroup	0.52 (0.48-0.56)	2	0.21	0.86	5.5	5.3
RR > 20	SAE during monitoring	0.60 (0.47-0.72)	16	0.81	0.48	168	153
RR > 21	SAE during monitoring	0.60 (0.47-0.72)	8	0.79	0.48	132	123
RR > 27	SAE after monitoring	0.51 (0.47-0.55)	2	0.25	0.78	11	17
SpO2 < 86	All patients	0.54 (0.48-0.60)	14	0.34	0.73	34	25
SpO2 < 93	Surgical subgroup	0.53 (0.46-0.60)	141	0.62	0.46	242	226
SpO2 < 87	Medical subgroup	0.60 (0.45-0.75)	58	0.52	0.66	93	72
SpO2 < 80	SAE during monitoring	0.63 (0.48-0.79)	2	0.38	0.86	5.6	5.6
SpO2 < 86	SAE after monitoring	0.54 (0.47-0.60)	14	0.32	0.75	30	23
SpO2 < 87	COPD	0.54 (0.41-0.67)	58	0.42	0.68	86	73
SpO2 < 88	COPD	0.54 (0.41-0.67)	28	0.65	0.50	111	92
SpO2 < 89	COPD	0.54 (0.41-0.67)	80	0.57	0.55	143	119
SpO2 < 86	Without COPD and BMI<40	0.53 (0.46-0.60)	42	0.15	0.92	24	16
SpO2 < 87	Without COPD and BMI<40	0.53 (0.46-0.60)	4	0.56	0.52	33	24
SpO2 < 92	Without COPD and BMI<40	0.53 (0.46-0.60)	50	0.72	0.36	173	160
SpO2 < 93	Without COPD and BMI<40	0.53 (0.46-0.60)	141	0.62	0.48	239	221

***Legend**: HR: heart rate; RR: respiratory rate; SpO2: peripheral oxygen saturation in %; SAE: serious adverse event; AUC: area under the curve; 95% CI: 95% confidence interval; COPD: chronic obstructive pulmonary disease; BMI: body mass index. Only thresholds with a cut-off <180 are presented.*

Number of patients in HR/RR analysis: total = 309, surgical subgroup = 250, medical subgroup = 59, SAE during monitoring = 42, SAE after monitoring = 267

Number of patients in SpO2 analysis: total = 176, surgical subgroup = 147, medical subgroup = 29, SAE during monitoring = 21, SAE after monitoring = 155, COPD = 40, without COPD and BMI<40 = 135

Table S7. Prognostic ability of optimal thresholds for SAE groups when comparing measurements from the last 12 hours before an SAE to those measured 12–24 hours prior.

Threshold	SAE group	Population	n SAE	AUC (95%CI)	Cut-off	Sensitivity	Specificity	Avg. time outside threshold (0-12 hours prior to SAE)	Avg. time outside threshold (12-24 hours prior to SAE)
SpO2 < 81	Infectious SAE	COPD	7	0.87 (0.68-1.00)	4	0.57	1.00	4.3	0.9
SpO2 < 83	Cardiovascular SAE	SAE during monitoring	6	0.86 (0.58-1.00)	2	1.00	0.83	39	4.0
RR > 18	Cardiovascular SAE	SAE during monitoring	8	0.81 (0.59-1.00)	6	1.00	0.62	106	45
SpO2 < 83	Infectious SAE	Medical subgroup	4	0.81 (0.50-1.00)	8	0.50	1.00	6.8	2.2
SpO2 < 87	All-cause mortality	Medical subgroup	4	0.75 (0.35-1.00)	69	0.75	0.75	92	56
HR > 102	Cardiovascular SAE	SAE during monitoring	8	0.73 (0.47-1.00)	2	0.75	0.75	30	7.9
SpO2 < 76	Respiratory SAE	SAE during monitoring	5	0.70 (0.30-1.00)	2	0.80	0.80	3.8	7.4

Legend: *HR: heart rate; RR: respiratory rate; SpO2: peripheral oxygen saturation in %; SAE: serious adverse event; n: number; AUC: area under the curve; 95% CI: 95% confidence interval; COPD: chronic obstructive pulmonary disease. Only thresholds with a cut-off <180 are presented*
